# Basal ganglia volume and shape in anorexia nervosa

**DOI:** 10.1016/j.appet.2019.104480

**Published:** 2020-01-01

**Authors:** Jenni Leppanen, Valentina Cardi, Felicity Sedgewick, Janet Treasure, Kate Tchanturia

**Affiliations:** a Kings' College London, Institute of Psychiatry, Psychology, and Neuroscience, Psychological Medicine, London, United Kingdom; b University of Bristol, 35 Berkeley Square, Clifton, Bristol, United Kingdom; c South London and Maudsley NHS Foundation Trust, London, United Kingdom; d Illia State University, Department of Psychology, Tbilisi, Georgia

**Keywords:** Basal ganglia, Pallidum, Caudate, Nucleus accumbens, Reward

## Abstract

**Background:**

Reward-centred models have proposed that anomalies in the basal ganglia circuitry that underlies reward learning and habit formation perpetuate anorexia nervosa (AN). The present study aimed to investigate the volume and shape of key basal ganglia regions, including the bilateral caudate, putamen, nucleus accumbens (NAcc), and globus pallidus in AN.

**Methods:**

The present study combined data from two existing studies resulting in a sample size of 46 women with AN and 56 age-matched healthy comparison (HC) women. Group differences in volume and shape of the regions of interest were examined. Within the AN group, the impact of eating disorder characteristics on volume and shape of the basal ganglia regions were also explored.

**Results:**

The shape analyses revealed inward deformations in the left caudate, right NAcc, and bilateral ventral and internus globus pallidus, and outward deformations in the right middle and posterior globus pallidus in the AN group.

**Conclusions:**

The present findings appear to fit with the theoretical models suggesting that there are alterations in the basal ganglia regions associated with habit formation and reward processing in AN. Further investigation of structural and functional connectivity of these regions in AN as well as their role in recovery would be of interest.

## 1 Introduction

Anorexia nervosa (AN) is a complex eating disorder characterised by severe malnutrition and relentless pursuit of thinness (American Psychiatric Association, 2013). The mortality rate in AN is one of the highest among psychiatric disorders (Arcelus, Mitchell, Wales, & Nielsen, 2011; Papadopoulos, Ekbom, Brandt, & Ekselius, 2009) and treatment remains a significant challenge (Steinhausen, 2002, 2009). Shedding light onto the processes and mechanisms that perpetuate AN is therefore of interest. Current theoretical models of AN have proposed that anomalies in reward motivation and learning may play a key role in the maintenance of disordered eating (Kaye, Frank, Bailer, & Henry, 2005; Kaye, Fudge, & Paulus, 2009; Keating, Tilbrook, Rossell, Enticott, & Fitzgerald, 2012; O'Hara, Campbell, & Schmidt, 2015). Most recently, the reward-centred model of AN postulates that early on weight loss may be met with positive social and emotional consequences, such as admiration and approval, while weight gain may be met with negative appraisal, increasing the reward associated with caloric restriction (O'Hara et al., 2015). Over time the reward motivation associated with weight loss cues increases, and so the associated eating disorder behaviours become compulsive and are repeated despite aversive consequences, such as negative affect, social isolation, and poor physical health. Thus, these theoretical models highlight similarities between AN and addiction, both of which are considered to be disorders of compulsivity and share a number of characteristics such as obsessionality and preoccupation (Crane, Roberts, & Treasure, 2007; Fontenelle, Oostermeijer, Harrison, Pantelis, & Yücel, 2011; Lubman, Yücel, & Pantelis, 2004; Serpell, Livingstone, Neiderman, & Lask, 2002).

Along with prefrontal and motor cortices, basal ganglia circuitry, including the globus pallidus and striatal regions, facilitates and supports reward-motivated learning and habitual responding (Ashby, Turner, & Horvitz, 2010; Balleine, Delgado, & Hikosaka, 2007). Preclinical studies have reported that reward-motivated decision making and acquisition of new learned behaviours depend on striatal regions, such as the caudate, anterior putamen, and nucleus accumbens (NAcc) (Goto & Grace, 2005; Gruber, Hussain, & O'Donnell, 2009; Yin, Ostlund, & Balleine, 2008). Temporary deactivation or permanent destruction of these regions impedes the acquisition of new rewarded actions, but does not negatively impact execution of previously learned habitual behaviours (Salamone, Correa, Farrar, & Mingote, 2007; Schultz, 2016). The caudate, anterior putamen, and NAcc are also sensitive to devaluation treatments, such as reduction in the expected value of outcome, and support extinction of non-rewarded actions (Izquierdo & Jentsch, 2012; Trifilieff et al., 2013). The posterior putamen and globus pallidus, on the other hand, have been proposed to facilitate formation of rigid habits and automatic responses, which are resistant to devaluation treatments and persist irrespective of consequences (Agustín-Pavón, Martínez-García, & Lanuza, 2014; McFarland & Kalivas, 2001; Saga et al., 2016; Sommer, Costa, & Hansson, 2014). These preclinical findings are in line with results from human studies (Boisgontier et al., 2016; Jahanshahi, Obeso, Rothwell, & Obeso, 2015; Tricomi, Balleine, & O'Doherty, 2009), which has sparked a great deal of interest in exploration of these regions in disorders of compulsivity.

Anomalies in the above mentioned basal ganglia circuitry are believed to play a key role in the maintenance of AN (O'Hara et al., 2015). In preclinical studies, mice exhibiting activity-based anorexia (ABA) show reduced metabolism in regions associated with reward-motivated learning, including the anterior parts of the caudate, NAcc, and putamen (Barbarich-Marsteller, Marsteller, Alexoff, Fowler, & Dewey, 2005; van Kuyck et al., 2007). Furthermore, ABA rats show greater resistance to extinction of food aversion than control rats (Liang, Bello, & Moran, 2011). Similarly, human studies have documented reduced regional blood flow and metabolism in the caudate in people with acute AN compared to healthy comparison individuals (Gaudio, Wiemerslage, Brooks, & Schiöth, 2016; Phillipou, Rossell, & Castle, 2014) while weight restored AN participants show reduced regional blood flow in the posterior putamen (Kojima et al., 2005). Furthermore, studies investigating structural differences in these regions have documented grey matter reduction in the caudate, NAcc, and putamen in acute AN (Friederich et al., 2012; Phillipou et al., 2018; Titova, Hjorth, Schiöth, & Brooks, 2013). A few studies have also investigated structural anomalies in weight restored AN participants and have found reduced globus pallidus volume and increased grey matter volume in the putamen, NAcc, and caudate (Bernardoni et al., 2016; Friederich et al., 2012). Taken together, these findings appear to mirror to a degree the preclinical findings detailed above and lend support to the notion that eating disorder related behaviours in AN may be habitual and compulsive, and are pursued despite negative consequences.

To our knowledge no studies to date have examined differences in the subcortical shape of the above mentioned basal ganglia circuitry in people with AN. While volumetric analysis can provide information about the overall size of a subcortical structure, vertex-wise shape-based analyses can detect alterations in the shape of subcortical structures and provides more information about regional anomalies in subcortical grey matter (Patenaude, Smith, Kennedy, & Jenkinson, 2011). Moreover, relative to other regional methods such as voxel-wise morphometry (VBM), vertex analysis provides more localised information about the geometric shape of the structure that is not sensitive to tissue-type segmentation or differences in prior smoothing (Patenaude et al., 2011). Therefore, examination of the shape of the basal ganglia circuitry in AN may be of interest. Indeed, a few recent studies have reported inward deformations in the shape of the caudate, NAcc, and anterior putamen and outward deformations in the posterior putamen and globus pallidus in disorders of compulsivity, including addiction, obsessive compulsive disorder (OCD), and trichotillomania (Choi et al., 2007; Garza-Villarreal et al., 2017; Isobe et al., 2018).

The aim of the present study was to explore the morphometry of basal ganglia regions including the bilateral caudate, putamen, globus pallidus, and NAcc in light of the reward-centred model of AN (O'Hara et al., 2015). In addition to volumetric analysis we examined differences between people with AN and healthy individuals in the shape of these regions. To examine the volume and shape of these structures and increase statistical power, anatomical neuroimaging data was combined from two studies (Fonville, Giampietro, Williams, Simmons, & Tchanturia, 2014; Leppanen et al., 2017a). Based on previous structural findings outlined above, we hypothesised that people with AN would show generally reduced volume of the caudate, NAcc, and putamen. We also hypothesised that participants with AN would have inward deformations in the shape of these regions that could provide further information about more localised atrophy. Based on the preclinical findings and vertex-wise shape findings from other disorders of compulsivity, we also hypothesised that people with AN may show greater outward deformation in regions associated with habitual responding, namely the posterior putamen and globus pallidus. Within the AN sample, we also conducted additional exploratory analyses to examine correlations between eating disorder characteristics, including body mass index (BMI), eating disorder psychopathology, and duration of illness, on subcortical volumes and shapes.

## 2 Materials and methods

### 2.1 Participants

The present study combined data from two previous studies (Fonville et al., 2014a, Fonville et al., 2014b; Leppanen et al., 2017a) conducted between 2011 and 2014. After removing repeat scans from 12 participants who had taken part in both studies, the combined sample consisted of 118 unique adult female participants over the age of 18, which included 56 participants with current DSM-IV diagnosis of AN (amenorrhea not required) and 62 healthy comparison (HC) participants. Fifteen participants were excluded due to either substantial missing data or left-handedness. Another three AN participants were excluded for having BMI over 18.5 as there was uncertainty regarding whether these participants were weight restored or long-term recovered at the time. The final sample consisted of 100 right handed women. Forty-six participants had current diagnosis of AN, which was confirmed using the Structured Clinical interview for Diagnosis – Researcher version (First, Williams, Karg, & Spitzer, 2015). The AN participants were recruited from the South London and Maudsley specialist eating disorders service and through online advertisements (BEAT eating disorders charity). Twenty-three AN participants reported taking psychotropic medication during the time of the studies. Further information about the type of psychotropic medication the participants were taking was only collected as part of one of the studies and was available for 13 of the medicated AN participants (Supplementary Table 1). Fifty-four participants formed an age-matched HC group with no current or history of psychiatric disorders, which was confirmed using the SCID-R. The HC participants were recruited from the local community and King's College London students and staff.

Participants were excluded if they reported acute suicidality, current or history of drug or alcohol misuse/abuse, any neurological disorders, or head trauma. Additionally, participants were excluded if they reported any MRI incompatibility, including pregnancy, any irremovable metal in or on the body, or claustrophobia. Prior to taking part all participants gave written informed consent and the two studies were approved by National Research Ethics Committees (11-LO-0952, 11/LO/0373). Both studies had prior ethical approval to use data later for further analysis. All research activities were conducted in accordance with the latest version of the Declaration of Helsinki (2013).

### 2.2 Procedure

The procedures in both studies were similar. In both studies all participants came to the King's College London Centre for Neuroimaging Sciences to undergo magnetic resonance imaging (MRI). Prior to the MRI, participants' height and weight were measured to calculate body mass index (BMI). Twenty-six women with current DSM-5 diagnosis of AN and 31 HC women took part in Study 1 between the years 2011 and 2014. In Study 1, the MRI session included a high resolution anatomical scan followed by three tasks which included two implicit facial emotion tasks reported elsewhere (Leppanen et al., 2017a; 2017b) and passive viewing of food and non-food images (Cardi, Leppanen, Mataix-Cols, Campbell, & Treasure, 2018). Thirty-six women with current DSM-5 diagnosis of AN and 37 HC women took part in Study 2, which was conducted between the years 2011 and 2013. In Study 2, the MRI session included a high resolution anatomical scan followed by an implicit facial emotion task, embedded figures test, and the Wisconsin Card Sorting Test reported elsewhere (Fonville et al., 2013; Fonville, Giampietro, Surguladze, Williams, & Tchanturia, 2014; Lao-Kaim et al., 2015). Although a whole brain VBM examination using the data from Study 2 has been published (Fonville et al., 2014a, Fonville et al., 2014b), the present study combined data from Study 2 with unpublished structural data from Study 1 and focused on vertex and volumetric analysis of specific, theory-driven regions of interest. Therefore, the present study should not constitute re-reporting published findings.

As part of both studies participants were asked to complete self-report questionnaires providing their age and duration of illness (in years). Participants were also asked to complete the Eating Disorder Examination Questionnaire (EDEQ), a validated 36-item self-report assessment of eating disorder symptomatology over the past 28 days (Fairburn & Beglin, 1994), and a self-report questionnaire assessing current levels of anxiety and depression. The two studies used different questionnaires to assess anxiety and depression. In Study 1, participants were asked to complete the Depression, Anxiety and Stress Scale (DASS), which is a 21-item self-report measure assessing the level of depression, anxiety, and stress over the past 2 weeks (Lovibond & Lovibond, 1995). In Study 2, participants completed the Hospital Anxiety and Depression Scale (HADS), which is a 14-item self-report instrument assessing level of depression and anxiety over the past week (Zigmond & Snaith, 1983). The internal consistency of the EDEQ (Cronbach's alpha = 0.96), DASS (Cronbach's alpha = 0.97), and HADS (Cronbach's alpha = 0.96) were high. To enable group comparisons across the two datasets, anxiety and depression subscales from the DASS and HADS were converted into z-scores. There was some missing data in the self-report measures in both studies: three AN participants did not report their exact age, 5 AN participant did not report their duration of illness, and one AN and one HC participant did not complete the EDEQ.

Data from Study 1 and Study 2 will be henceforth be referred to as dataset 1 and dataset 2, respectively.

### 2.3 Image acquisition

Both studies used the same MRI scanner unit housed at the King's College London, Centre for Neuroimaging Sciences. The anatomical MRI was conducted with GE Signa 1.5T scanner unit (GE Medical Systems, Milwaukee, Wisconsin). Both studies used the same scanning parameters to acquire the T1-weighted anatomical magnetization-prepared rapid gradient-echo (MP-RAGE) images. The MP-RAGE images were acquired with 1.2 mm slice thickness, 1.2 mm slice gap, 8° flip angle, 8.59 s repetition time, 3.8 s echo time, and voxel size of 1.2mmx1.2 mm × 1.2  mm. Full brain coverage was achieved with 180 sagittal slices and an 8-channel headcoil was used to transmit and receive the signal.

### 2.4 Statistical analysis

#### 2.4.1 Clinical and demographic data analysis

All clinical and demographic data were analysed using R (R Core Team, 2018). Group differences in age, BMI, EDEQ, total score and anxiety and depression z-scores were examined using between-subjects t-tests. P < 0.05 was considered significant.

#### 2.4.2 Imaging data analysis

All anatomical images were analysed with FSL (v5.0.11). The images were first preprocessed using *run_first_all*, which includes brain extraction, segmentation, formation of subcortical mesh and volumetric outputs, and boundary correction (https://fsl.fmrib.ox.ac.uk/fsl/fslwiki/FIRST/UserGuide). Following preprocessing, we used the *first_roi_slicesdir* function to generate summary images, which were visually inspected for segmentation errors by two authors (J.L. and F.S.), who were both blind to the diagnostic group each scan belonged to. Both authors inspected all summary images. Any images that showed evidence of poor segmentation according to either author were further assessed to find the cause of the segmentation error followed by re-running of the *run_first_all* function. The preprocessing and quality assessment procedures was repeated for four of the images due to segmentation errors that arose from coordinate mismatch.

Information regarding volume (mm^3^) of the subcortical regions of interest, namely the bilateral caudate, putamen, globus pallidus, and NAcc, were acquired using *fslstats*. Subcortical volumes were then entered into R (R Core Team, 2018) for statistical analysis. Group differences controlling for dataset were examined with multiple linear regressions (*lm*). Correlations between subcortical volumes and BMI, EDEQ total score, and duration of illness were explored. Prior to group comparisons and correlation analyses, differences in subcortical volume between the two datasets were examined. There were no significant differences between the two datasets (Supplementary Table 2). Thus, both datasets were analysed together. Still, dataset was entered as a discrete nuisance covariate in all vertex analyses, to ensure no group differences or correlations were present due to potential small differences between the two datasets. False Discovery Rate correction with q = 0.05 was used to adjust the p-threshold for multiple comparisons and p < 0.003 was considered significant.

Vertex-wise subcortical shape analysis was conducted with FSL (v5.0.11) using *first_utils* (https://fsl.fmrib.ox.ac.uk/fsl/fslwiki/FIRST/UserGuide). Each participant's vertex-wise shape statistics are projected onto a group average surface and all meshes were reconstructed in Montreal Neurological Institute (MNI) space using rigid alignment transformation with 6 degrees of freedom. Negative vertex-wise values indicate inward deformation in the shape of the subcortical structure, while positive values indicate outward deformation. Differences between the AN and HC groups were conducted using a non-parametric permutation test with 5000 permutations (*randomise*). Additional exploratory analyses were conducted examining correlations between vertex-wise values and BMI, eating disorder symptomatology, and duration of illness within the AN group. All results were corrected for multiple comparisons using the threshold-free cluster enhancement (TFCE) method (Smith & Nichols, 2009), which identifies clusters by enhancing voxels where the signal shows spatial contiguity. All p-values reported in the subcortical shape analysis section below are TFCE corrected p-values and p < 0.05 was considered significant. Prior to group comparisons and correlation analyses, differences in subcortical shape between the two datasets were examined. There were no significant differences between the two datasets in subcortical shape. Thus, both datasets were analysed together. Still, dataset was entered as a discrete nuisance covariate in all vertex analyses, to ensure no group differences or correlations were present due to potential small differences between the two datasets.

## 3 Results

### 3.1 Demographic and clinical characteristics

Demographic and clinical characteristics by group are presented in Table 1. The groups were matched for age. As expected there was a significant difference in BMI, EDEQ total score, anxiety z-score, and depression z-score such that the AN group had lower BMI and reported more eating disorder symptomatology, anxiety, and depression than the HC group.

**Table 1:** Demographic and clinical characteristics.

	AN M (SD)	HC M (SD)	t score, p-value
Age	27.51 (9.24)	26.35 (4.47)	t(58) = 0.76, p = 0.453
EDEQ Total	4.01 (1.01)	0.54 (0.51)	t(62) = 20.79, p < 0.001
Anxiety z-score	0.90 (0.67)	−0.77 (0.39)	t(69) = 14.88, p < 0.001
Depression z-score	0.85 (0.83)	−0.73 (0.31)	t(56) = 12.18, p < 0.001
BMI	15.73 (1.41)	21.49 (1.97)	t(95) = −16.96, p < 0.001
Duration of illness (years)	11.39 (9.22)	N/A	N/A

AN = anorexia nervosa, HC = healthy comparison, EDEQ = Eating Disorder Examination Questionnaire, BMI = body mass index, M = mean, SD = standard deviation, N/A = not applicable.

### 3.2 Subcortical volume

#### 3.2.1 Differences between AN and HC groups

Subcortical volumes by group are presented in Table 2. Following correction for multiple comparisons, there were no significant differences between AN and HC group in subcortical volume controlling for dataset. There was also no significant difference between the two datasets across groups.

**Table 2:** Subcortical volume by group.

Hemisphere	Volume	AN M (SD)	HC M (SD)	t score, p value
left	Caudate	3595.82 (368.18)	3668.41 (347.67)	Group: t(97) = 0.98, p = 0.330 Dataset: t(97) = −1.83, p = 0.071
	Putamen	4383.92 (443.59)	4437.08 (534.02)	Group: t(97) = 0.53, p = 0.597 Dataset: t(97) = −0.08, p = 0.935
	Globus pallidus	1322.97 (166.51)	1374.80 (173.03)	Group: t(97) = 1.50, p = 0.138 Dataset: t(97) = −0.77, p = 0.442
	NAcc	577.37 (84.95)	589.00 (124.63)	Group: t(97) = 0.52, p = 0.603 Dataset: t(97) = −0.45, p = 0.653
Right	Caudate	3620.95 (411.63)	3693.41 (336.76)	Group: t(97) = 0.93, p = 0.353 Dataset: t(97) = −1.93, p = 0.057
	Putamen	4204.35 (452.18)	4270.18 (523.61)	Group: t(97) = 0.67, p = 0.507 Dataset: t(97) = 0.12, p = 0.907
	Globus pallidus	1458.66 (171.20)	1432.12 (171.36)	Group: t(97) = −0.79, p = 0.432 Dataset: t(97) = −0.78, p = 0.438
	NAcc	486.46 (72.42)	524.30 (113.30)	Group: t(97) = 1.96, p = 0.053 Dataset: t(97) = 0.56, p = 0.577

AN = anorexia nervosa, HC = healthy comparison, NAcc = Nucleus accumbens, M = mean, SD = standard deviation.

#### 3.2.2 Correlations with clinical characteristics

Correlations between BMI, EDEQ total score and duration of illness, and subcortical volumes are presented in Supplementary Table 3. Following correction for multiple comparisons, there were no significant correlations between eating disorder characteristics and subcortical volumes.

### 3.3 Subcortical shape

#### 3.3.1 Differences between AN and HC groups

**Fig. 1:**
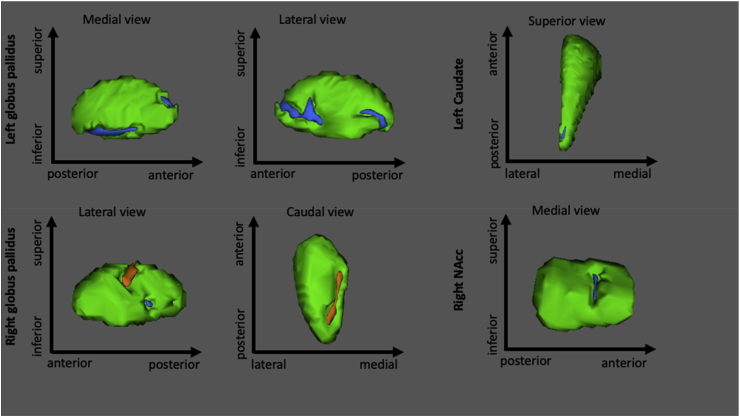
Group differences in subcortical shape.

Group differences in subcortical shape controlling for dataset are presented in Table 3 and Fig. 1. In the left hemisphere, the AN group had significantly smaller vertex indices in the caudate (t = −4.44, p = 0.022) and globus pallidus (C1: t = −5.16, p = 0.001; C2: t = −4.97, p = 0.004) relative to the HC group. In the right hemisphere, the AN group had smaller vertex indices in the NAcc (t = −3.52, p = 0.027) and in the right globus pallidus (C3: t = −4.33, p = 0.025) relative to the HC group. The AN group also had significantly greater vertex indices in two clusters in the right globus pallidus (C1: t = 3.63, p = 0.027; C2: t = 4.11, p = 0.013) compared to the HC group.

**Table 3:** Group differences in subcortical shape.

Hemisphere	Structure	Index	Voxels	Peak MNI coordinates			Peak F score, TFCE corrected p-value
				X	Y	Z	
Left	Caudate	C1	16	−19	−18	25	F = 9.85, p = 0.027
	Globus pallidus	C1	100	−19	2	−6	F = 13.30, p = 0.001
		C2	67	−18	−10	−3	F = 12.30, p = 0.003
Right	NAcc	C1	5	10	15	−4	F = 6.18, p = 0.041
	Globus pallidus	C1	27	17	−3	3	F = 8.46, p = 0.015
		C2	23	24	−11	−6	F = 6.33, p = 0.032
		C3	12	18	−8	−3	F = 9.37, p = 0.026

TFCE = threshold-free cluster enhancement, NAcc = Nucleus accumbens, MNI = Montreal Neurological Institute.

The blue clusters indicate smaller vertices in the AN group relative to the HC group and the orange colour indicated greater vertices in the AN group relative to the HC group. NAcc = Nucleus accumbens.

#### 3.3.2 Correlations with clinical characteristics

There were no significant correlations between BMI, EDEQ total score, or duration of illness and vertex indices within the AN group. Within the HC group, there was a negative correlation between BMI and surface deformations in the anterior and lateral parts of the left putamen (Supplementary Table 4). There were no other significant correlations between vertex indices and BMI or EDEQ total score within the HC group.

## 4 Discussion

The aim of the present study was to investigate differences in the volume and shape of basal ganglia structures hypothesised to play a key role in the maintenance of AN (O'Hara et al., 2015). Contrary to our hypotheses we did not find significant differences between AN and HC participants in left or right caudate, putamen, globus pallidus, or NAcc volume. However, as hypothesised, there was evidence of localised anomalies in the shape of the left caudate, bilateral globus pallidus and right NAcc in the AN group. In our exploratory correlation analyses we did not find any significant correlations between subcortical volume or shape and eating disorder characteristics.

The present findings partly support and partly contradict our hypotheses showing inward deformations in the left caudate body and right NAcc, but also in the left ventral and internus globus pallidus, and right internus globus pallidus. These findings appear to suggest that the globus pallidus is a complex structure and may facilitate many function, not only habitual, rigid stimulus-response actions. Similar inward deformations in the left caudate body and right NAcc have been found in trichotillomania and crack cocaine addiction (Garza-Villarreal et al., 2017; Isobe et al., 2018). Such inward deformation of the striatum have been suggested as being linked to reward evaluation and reward-motivated decision making, possibly suggesting that such weighing of behaviour and outcome may disrupted in disorders of compulsion. Moreover, pathways linking the striatum to the ventral globus pallidus have been proposed to play a role in controlling behaviour (Aouizerate et al., 2004; Gillan et al., 2015; Narayanaswamy, Jose, Kalmady, Venkatasubramanian, & Reddy, 2013). Functional anomalies in this striato-pallidal pathway have been suggested to underlie repetitive, pathological behaviours in OCD (Aouizerate et al., 2004; Beucke et al., 2013). It has been suggested that this pathway fails to signal for the end of a behavioural routine, leading to a pathological loop which maintains the compulsive disorder-related behaviour (Aouizerate et al., 2004). As compulsivity is feature that has been linked to both OCD and AN (Montigny et al., 2013) and strong genetic correlation has recently been reported between AN and OCD (Watson et al., 2019), similar striato-pallidal functional anomalies may be present in AN. Thus, further investigation of potential anomalies in the functional and structural pathways that link these regions as well as the role they may play in the maintenance of eating disorder behaviours may be of interest.

As hypothesised the present study revealed evidence of outward deformations in the middle and posterior internus globus pallidus in the AN participants. Similar outward deformations as well as increased grey matter in the middle and posterior internus globus pallidus have been documented in OCD (Shaw et al., 2015; Zarei et al., 2011). Furthermore, atypical increased activation in the globus pallidus and connected regions in the striatum, thalamus, and frontal cortex have also been reported to be linked to compulsive behaviours in OCD (Rotge et al., 2008). These findings appear to fit well with preclinical findings reporting that the posterior parts of the globus pallidus has a key role supporting and facilitating habitual, rigid behaviours (Agustín-Pavón et al., 2014; McFarland & Kalivas, 2001; Saga et al., 2016; Sommer et al., 2014). Therefore, further investigation of the functional connectivity of the posterior globus pallidus and its role in illness maintenance in AN would be of interest.

Our exploratory correlation analyses did not reveal any significant relationships between basal ganglia volumes or shapes and eating disorder characteristics. Some previous studies have reported significant relationships global grey matter and BMI in people with AN as well as between BMI and grey matter increase following weight restoration in AN (Mainz, Schulte-Rüther, Fink, Herpertz-Dahlmann, & Konrad, 2012; Mühlau et al., 2007). However, many previous studies have also failed to find significant correlation between bran structure and self-reported clinical variables among people with AN (Boghi et al., 2011; Brooks et al., 2011; Frank, Shott, Hagman, & Mittal, 2013; Mühlau et al., 2007; Suchan et al., 2010), possibly highlighting the difficulty of using self-report measures. Future studies may benefit from conducting longitudinal examinations to clarify the potential role of structural and functional anomalies in these regions have perpetuating the illness.

### 4.1 Limitations

The present study is not without limitations. Firstly, we did not have a behavioural measure to assess reward processing, learning, habit formation, or compulsivity. The use of such measures would be necessary to confirm that the present basal ganglia deformations are linked to reward learning and compulsivity. Therefore, future studies may benefit from incorporating such behavioural measures and neuroimaging to further explore this potential link in AN as well as its role in illness maintenance or recovery in a longitudinal setting.

The present study investigated the volume and shape basal ganglia regions, but not their connections. Therefore, it is difficult to ascertain how the shape anomalies found may be linked to each other. Future studies may benefit from further exploring structural connections and pathways between these regions. A few studies have begun to investigate structural pathways in AN using diffusion tensor imaging (DTI) and have reported anomalies in white matter track in a number of regions including the corpus callosum and cingulum, but findings are still somewhat mixed (King, Frank, Thompson, & Ehrlich, 2018). Thus, further examination of the basal ganglia regions hypothesised to play a role in the maintenance of AN using DTI techniques may be of interest.

It is also of importance to note that structural differences do not necessarily indicate that there are any functional differences. Indeed, several studies that have investigated both brain structure and function have reported structural group differences in regions that have shown no functional group differences (Anurova, Renier, De Volder, Carlson, & Rauschecker, 2015; Pereira et al., 2018; Tavor et al., 2016). One study found that less than 3% of the maximum voxels in voxel-based morphometry analysis matched those from a resting-state functional connectivity analysis (Pereira et al., 2018). Furthermore, studies attempting to map functional and structural connectivity studies investigating correlations between structural and functional MRI have found that although there are some links between resting state functional connectivity and structural pathways, direct functional connectivity has also been found between regions that show no direct structural connectivity (Damoiseaux & Greicius, 2009; Honey et al., 2009). Therefore, before firm conclusion regarding the role of basal ganglia regions in the maintenance of eating disorder behaviours in AN are drawn the function of these regions in relation to illness related variables should be examined.

Although many recent studies have used a 3.0T MRI scanner unit, the present study used data acquired with a 1.5T MRI unit. The increased filed strength of a 3.0T MRI unit has been suggested to improve signal-to-noise ratio leading to higher quality anatomical images (Takahashi, Uematsu, & Hatabu, 2003). A few studies comparison 1.5T and 3.0T MRI scanner units have reported that the 3.0T unit has greater sensitivity, particularly in detecting small scale structural anomalies in neurological disorders such as multiple sclerosis and Alzheimer's disease (Chow et al., 2015; Stankiewicz et al., 2011). 3.0T MR images have also been found to provide more clinically relevant information than images acquired using a 1.5T MRI unit during pre-surgical review (Bingaman et al., 2004). However, a recent systematic review reported that the 1.5T and 3.0T units were equivalent and although differences were found they were largely too divergent to conclude that one was superior to the other (Wardlaw et al., 2012). This finding could explained by findings that improved signal-to-noise ratio at higher field strength may be offset by failure to take other differences into consideration such as increased T1 relaxation time (Takahashi et al., 2003). Thus, although using data acquired with a lower field strength MRI unit may have affected the present findings, the extent of the effect is unclear.

It is uncertain to what extent and how the use of psychotropic medication impacted the present findings. A systematic review found that psychotropic treatment was associated with both structural and functional changes in a number of brain regions including the basal ganglia, in bipolar disorder, schizophrenia, and attention deficit with hyperactivity disorder, which in turn were associated with symptom improvement (Singh & Chang, 2012). In the present study we were unable to examine the impact of medication on basal ganglia volume and shape. Information regarding the type of medication participants were taking when the images were acquired was only collected as part of one study. Additionally, when this information was available it was clear that many participants were taking many different types of medication, which likely introduced heterogeneity to any investigation of impact of medication of basal ganglia volume and shape. Furthermore, information regarding how long participants had been taking psychotropic medication was not collected as part of either study. Future studies may benefit from controlling for the types of medication participants are taking in order to examine the impact of psychotropic medication of basal ganglia volume and shape.

Finally, as the present study was cross-sectional in nature it is not possible to ascertain to what extent the findings may be related to state of malnutrition in AN (Phillipou et al., 2018). Furthermore, duration of illness, information regarding psychotropic medication, and eating disorder symptomatology were assessed through self-report. Thus, these measures could have been affected by uncertainty regarding the exact onset of illness and lack of insight into the illness. Additionally, we did not have sufficient information to regarding AN subtype to investigate differences between restricting and binge/purge AN participants. Future studies may benefit from linking with clinicians to gain information about illness subtype and to corroborate self-report measures with clinician report.

### 4.2 Conclusions

Reward-centred theoretical models postulate that anomalies in the basal ganglia circuitry that underlies reward processing, learning, and habit formation have a key role in the maintenance of AN. The aim of the present study was to investigate the volume and shape of key basal ganglia regions including bilateral caudate, putamen, NAcc, and globus pallidus in women with and without AN. The study combined data from two existing studies resulting in a sample size of 46 women with AN and 56 HC women. There were no significant differences between the groups in the volume of any of the regions of interest. However, there were small, localised group differences in the shape of these regions. The results revealed areas of inward deformations in the AN group relative to the HC group in the left caudate, right NAcc, and bilateral globus pallidus. Additionally there were small areas of outward deformation in the AN group relative to the HC group in the right globus pallidus. These findings are in line with the reward-centred models of AN and future research may benefit from further investigation of the role of these regions in reward processing in AN as well as their potential role in the maintenance of the illness in long term would be of interest.

## Declaration of competing interest

None.

## References

[bib1] Agustín-Pavón C., Martínez-García F., Lanuza E. (2014). Focal lesions within the ventral striato-pallidum abolish attraction for male chemosignals in female mice. Behavioural Brain Research.

[bib2] American Psychiatric Association (2013). Diagnostic and statistical manual of mental disorders (DSM-5®).

[bib3] Anurova I., Renier L.A., De Volder A.G., Carlson S., Rauschecker J.P. (2015). Relationship between cortical thickness and functional activation in the early blind. Cerebral Cortex (New York, N.Y. : 1991).

[bib4] Aouizerate B., Guehl D., Cuny E., Rougier A., Bioulac B., Tignol J. (2004). Pathophysiology of obsessive–compulsive disorder: A necessary link between phenomenology, neuropsychology, imagery and physiology. Progress in Neurobiology.

[bib5] Arcelus J., Mitchell A.J., Wales J., Nielsen S. (2011). Mortality rates in patients with anorexia nervosa and other eating disorders. Archives of General Psychiatry.

[bib6] Ashby F.G., Turner B.O., Horvitz J.C. (2010). Cortical and basal ganglia contributions to habit learning and automaticity. Trends in Cognitive Sciences.

[bib7] Balleine B.W., Delgado M.R., Hikosaka O. (2007). The role of the dorsal striatum in reward and decision-making. Journal of Neuroscience.

[bib8] Barbarich-Marsteller N.C., Marsteller D.A., Alexoff D.L., Fowler J.S., Dewey S.L. (2005). MicroPET imaging in an animal model of anorexia nervosa. Synapse.

[bib9] Bernardoni F., King J.A., Geisler D., Stein E., Jaite C., Nätsch D. (2016). Weight restoration therapy rapidly reverses cortical thinning in anorexia nervosa: A longitudinal study. NeuroImage.

[bib10] Beucke J.C., Sepulcre J., Talukdar T., Linnman C., Zschenderlein K., Endrass T. (2013). Abnormally high degree connectivity of the orbitofrontal cortex in obsessive-compulsive disorder. JAMA Psychiatry.

[bib11] Bingaman W.E., Triantafyllou C., Wald L.L., Wiggins G., Kirk G.P., Larsson P.G. (2004). Surgery for focal cortical dysplasia. Neurology.

[bib12] Boghi A., Sterpone S., Sales S., D'Agata F., Bradac G.B., Zullo G. (2011). In vivo evidence of global and focal brain alterations in anorexia nervosa. Psychiatry Research: Neuroimaging.

[bib13] Boisgontier M.P., van Ruitenbeek P., Leunissen I., Chalavi S., Sunaert S., Levin O. (2016). Nucleus accumbens and caudate atrophy predicts longer action selection times in young and old adults. Human Brain Mapping.

[bib14] Brooks S.J., Barker G.J., O'Daly O.G., Brammer M., Williams S.C., Benedict C. (2011). Restraint of appetite and reduced regional brain volumes in anorexia nervosa: A voxel-based morphometric study. BMC Psychiatry.

[bib15] Cardi V., Leppanen J., Mataix-Cols D., Campbell I.C., Treasure J. (2018). A case series to investigate food-related fear learning and extinction using in vivo food exposure in anorexia nervosa: A clinical application of the inhibitory learning framework. European eating disorders review.

[bib16] Choi J.-S., Kim S.H., Yoo S.Y., Kang D.-H., Kim C.-W., Lee J.-M. (2007). Shape deformity of the corpus striatum in obsessive–compulsive disorder. Psychiatry Research: Neuroimaging.

[bib17] Chow, N., Hwang, K. S., Hurtz, S., Green, A. E., Somme, J. H., Thompson, P. M., … Alzheimer’s Disease Neuroimaging Initiative. (2015). Comparing 3T and 1.5T MRI for mapping hippocampal atrophy in the Alzheimer’s Disease Neuroimaging Initiative. AJNR. American Journal of Neuroradiology, 36(4), 653-660. 10.3174/ajnr.A4228. PMC5832348 25614473

[bib18] Crane A.M., Roberts M.E., Treasure J. (2007). Are obsessive-compulsive personality traits associated with a poor outcome in anorexia nervosa? A systematic review of randomized controlled trials and naturalistic outcome studies. International Journal of Eating Disorders.

[bib19] Damoiseaux J.S., Greicius M.D. (2009). Greater than the sum of its parts: A review of studies combining structural connectivity and resting-state functional connectivity. Brain Structure and Function.

[bib20] Fairburn C.G., Beglin S.J. (1994). Assessment of eating disorders: Interview or self-report questionnaire?. The International Journal of Eating Disorders.

[bib21] First M.B., Williams J.B.W., Karg R.S., Spitzer R.L. (2015). Structured clinical interview for DSM-5, research version (SCID-5-RV).

[bib22] Fontenelle L.F., Oostermeijer S., Harrison B.J., Pantelis C., Yücel M. (2011). Obsessive-compulsive disorder, impulse control disorders and drug addiction. Drugs.

[bib23] Fonville L., Giampietro V., Surguladze S., Williams S., Tchanturia K. (2014). Increased BOLD signal in the fusiform gyrus during implicit emotion processing in anorexia nervosa. NeuroImage: Clinical.

[bib24] Fonville L., Giampietro V., Williams S.C.R., Simmons A., Tchanturia K. (2014). Alterations in brain structure in adults with anorexia nervosa and the impact of illness duration. Psychological Medicine.

[bib25] Fonville L., Lao-Kaim N.P., Giampietro V., Van den Eynde F., Davies H., Lounes N. (2013). Evaluation of enhanced attention to local detail in anorexia nervosa using the embedded figures test; an fMRI study. PLoS One.

[bib26] Frank G.K., Shott M.E., Hagman J.O., Mittal V.A. (2013). Alterations in brain structures related to taste reward circuitry in ill and recovered anorexia nervosa and in bulimia nervosa. American Journal of Psychiatry.

[bib27] Friederich H.-C., Walther S., Bendszus M., Biller A., Thomann P., Zeigermann S. (2012). Grey matter abnormalities within cortico-limbic-striatal circuits in acute and weight-restored anorexia nervosa patients. NeuroImage.

[bib28] Garza-Villarreal E.A., Chakravarty M., Hansen B., Eskildsen S.F., Devenyi G.A., Castillo-Padilla D. (2017). The effect of crack cocaine addiction and age on the microstructure and morphology of the human striatum and thalamus using shape analysis and fast diffusion kurtosis imaging. Translational Psychiatry.

[bib29] Gaudio S., Wiemerslage L., Brooks S.J., Schiöth H.B. (2016). A systematic review of resting-state functional-MRI studies in anorexia nervosa: Evidence for functional connectivity impairment in cognitive control and visuospatial and body-signal integration. Neuroscience & Biobehavioral Reviews.

[bib30] Gillan C.M., Apergis-Schoute A.M., Morein-Zamir S., Urcelay G.P., Sule A., Fineberg N.A. (2015). Functional neuroimaging of avoidance habits in obsessive-compulsive disorder. American Journal of Psychiatry.

[bib31] Goto Y., Grace A.A. (2005). Dopaminergic modulation of limbic and cortical drive of nucleus accumbens in goal-directed behavior. Nature Neuroscience.

[bib32] Gruber A.J., Hussain R.J., O'Donnell P. (2009). The nucleus accumbens: A switchboard for goal-directed behaviors. PLoS One.

[bib33] Honey C.J., Sporns O., Cammoun L., Gigandet X., Thiran J.P., Meuli R. (2009). Predicting human resting-state functional connectivity from structural connectivity. Proceedings of the National Academy of Sciences of the United States of America.

[bib34] Isobe M., Redden S.A., Keuthen N.J., Stein D.J., Lochner C., Grant J.E. (2018). Striatal abnormalities in trichotillomania: A multi-site MRI analysis. NeuroImage: Clinical.

[bib35] Izquierdo A., Jentsch J.D. (2012). Reversal learning as a measure of impulsive and compulsive behavior in addictions. Psychopharmacology.

[bib36] Jahanshahi M., Obeso I., Rothwell J.C., Obeso J.A. (2015). A fronto–striato–subthalamic–pallidal network for goal-directed and habitual inhibition. Nature Reviews Neuroscience.

[bib37] Kaye W.H., Frank G.K., Bailer U.F., Henry S.E. (2005). Neurobiology of anorexia nervosa: Clinical implications of alterations of the function of serotonin and other neuronal systems. International Journal of Eating Disorders.

[bib38] Kaye W.H., Fudge J.L., Paulus M. (2009). New insights into symptoms and neurocircuit function of anorexia nervosa. Nature Reviews Neuroscience.

[bib39] Keating C., Tilbrook A.J., Rossell S.L., Enticott P.G., Fitzgerald P.B. (2012). Reward processing in anorexia nervosa. Neuropsychologia.

[bib40] King J.A., Frank G.K.W., Thompson P.M., Ehrlich S. (2018). Structural neuroimaging of anorexia nervosa: Future directions in the quest for mechanisms underlying dynamic alterations. Biological Psychiatry.

[bib41] Kojima S., Nagai N., Nakabeppu Y., Muranaga T., Deguchi D., Nakajo M. (2005). Comparison of regional cerebral blood flow in patients with anorexia nervosa before and after weight gain. Psychiatry Research: Neuroimaging.

[bib42] van Kuyck K., Casteels C., Vermaelen P., Bormans G., Nuttin B., Van Laere K. (2007). Motor- and food-related metabolic cerebral changes in the activity-based rat model for anorexia nervosa: A voxel-based microPET study. NeuroImage.

[bib43] Lao-Kaim N.P., Fonville L., Giampietro V.P., Williams S.C.R., Simmons A., Tchanturia K. (2015). Aberrant function of learning and cognitive control networks underlie inefficient cognitive flexibility in anorexia nervosa: A cross-sectional fMRI study. PLoS One.

[bib44] Leppanen J., Cardi V., Paloyelis Y., Simmons A., Tchanturia K., Treasure J. (2017). Blunted neural response to implicit negative facial affect in anorexia nervosa. Biological Psychology.

[bib45] Leppanen J., Cardi V., Paloyelis Y., Simmons A., Tchanturia K., Treasure J. (2017). FMRI study of neural responses to implicit infant emotion in anorexia nervosa. Frontiers in Psychology.

[bib46] Liang N.-C., Bello N.T., Moran T.H. (2011). Experience with activity based anorexia enhances conditioned taste aversion learning in rats. Physiology & Behavior.

[bib47] Lovibond P.F., Lovibond S.H. (1995). The structure of negative emotional states: Comparison of the depression anxiety stress scales (DASS) with the beck depression and anxiety inventories. Behaviour Research and Therapy.

[bib48] Lubman D.I., Yücel M., Pantelis C. (2004). Addiction, a condition of compulsive behaviour? Neuroimaging and neuropsychological evidence of inhibitory dysregulation. Addiction.

[bib49] Mainz V., Schulte-Rüther M., Fink G.R., Herpertz-Dahlmann B., Konrad K. (2012). Structural brain abnormalities in adolescent anorexia nervosa before and after weight recovery and associated hormonal changes. Psychosomatic Medicine.

[bib50] McFarland K., Kalivas P.W. (2001). The circuitry mediating cocaine-induced reinstatement of drug-seeking behavior. Journal of Neuroscience: The Official Journal of the Society for Neuroscience.

[bib51] Montigny C., Castellanos-Ryan N., Whelan R., Banaschewski T., Barker G.J., Büchel C. (2013). A phenotypic structure and neural correlates of compulsive behaviors in adolescents. PLoS One.

[bib52] Mühlau M., Gaser C., Ilg R., Conrad B., Leibl C., Cebulla M.H. (2007). Gray matter decrease of the anterior cingulate cortex in anorexia nervosa. American Journal of Psychiatry.

[bib53] Narayanaswamy J.C., Jose D., Kalmady S., Venkatasubramanian G., Reddy Y.J. (2013). Clinical correlates of nucleus accumbens volume in drug-naïve, adult patients with obsessive–compulsive disorder. Australian and New Zealand Journal of Psychiatry.

[bib54] O'Hara C.B., Campbell I.C., Schmidt U. (2015). A reward-centred model of anorexia nervosa: A focussed narrative review of the neurological and psychophysiological literature. Neuroscience & Biobehavioral Reviews.

[bib55] Papadopoulos F.C., Ekbom A., Brandt L., Ekselius L. (2009). Excess mortality, causes of death and prognostic factors in anorexia nervosa. British Journal of Psychiatry.

[bib56] Patenaude B., Smith S.M., Kennedy D.N., Jenkinson M. (2011). A Bayesian model of shape and appearance for subcortical brain segmentation. NeuroImage.

[bib57] Pereira A.M., Campos B.M., Coan A.C., Pegoraro L.F., de Rezende T.J.R., Obeso I. (2018). Differences in cortical structure and functional MRI connectivity in high functioning autism. Frontiers in Neurology.

[bib58] Phillipou A., Rossell S.L., Castle D.J. (2014). The neurobiology of anorexia nervosa: A systematic review. Australian and New Zealand Journal of Psychiatry.

[bib59] Phillipou A., Rossell S.L., Gurvich C., Castle D.J., Abel L.A., Nibbs R.G. (2018). Differences in regional grey matter volumes in currently ill patients with anorexia nervosa. European Journal of Neuroscience.

[bib60] R Core Team (2018). R: A language and environment for statistical computing. https://www.r-project.org/.

[bib61] Rotge J.-Y., Guehl D., Dilharreguy B., Cuny E., Tignol J., Bioulac B. (2008). Provocation of obsessive-compulsive symptoms: A quantitative voxel-based meta-analysis of functional neuroimaging studies. Journal of Psychiatry & Neuroscience: Journal of Psychiatry & Neuroscience.

[bib62] Saga Y., Richard A., Sgambato-Faure V., Hoshi E., Tobler P.N., Tremblay L. (2016). Ventral pallidum encodes contextual information and controls aversive behaviors. Cerebral Cortex.

[bib63] Salamone J.D., Correa M., Farrar A., Mingote S.M. (2007). Effort-related functions of nucleus accumbens dopamine and associated forebrain circuits. Psychopharmacology.

[bib64] Schultz W. (2016). Reward functions of the basal ganglia. Journal of Neural Transmission.

[bib65] Serpell L., Livingstone A., Neiderman M., Lask B. (2002). Anorexia nervosa: Obsessive–compulsive disorder, obsessive–compulsive personality disorder, or neither?. Clinical Psychology Review.

[bib66] Shaw P., Sharp W., Sudre G., Wharton A., Greenstein D., Raznahan A. (2015). Subcortical and cortical morphological anomalies as an endophenotype in obsessive-compulsive disorder. Molecular Psychiatry.

[bib67] Singh M.K., Chang K.D. (2012). The neural effects of psychotropic medications in children and adolescents. Child and Adolescent Psychiatric Clinics of North America.

[bib68] Smith S., Nichols T. (2009). Threshold-free cluster enhancement: Addressing problems of smoothing, threshold dependence and localisation in cluster inference. NeuroImage.

[bib69] Sommer W.H., Costa R.M., Hansson A.C. (2014). Dopamine systems adaptation during acquisition and consolidation of a skill. Frontiers in Integrative Neuroscience.

[bib70] Stankiewicz J.M., Glanz B.I., Healy B.C., Arora A., Neema M., Benedict R.H.B. (2011). Brain MRI lesion load at 1.5T and 3T versus clinical status in multiple sclerosis. Journal of Neuroimaging: Official Journal of the American Society of Neuroimaging.

[bib71] Steinhausen H.-C. (2002). The outcome of anorexia nervosa in the 20th century. American Journal of Psychiatry.

[bib72] Steinhausen H.-C. (2009). Outcome of eating disorders. Child and Adolescent Psychiatric Clinics of North America.

[bib73] Suchan B., Busch M., Schulte D., Grönermeyer D., Herpertz S., Vocks S. (2010). Reduction of gray matter density in the extrastriate body area in women with anorexia nervosa. Behavioural Brain Research.

[bib74] Takahashi M., Uematsu H., Hatabu H. (2003). MR imaging at high magnetic fields. European Journal of Radiology.

[bib75] Tavor I., Parker Jones O., Mars R.B., Smith S.M., Behrens T.E., Jbabdi S. (2016). Task-free MRI predicts individual differences in brain activity during task performance. Science (New York, N.Y.).

[bib76] Titova O.E., Hjorth O.C., Schiöth H.B., Brooks S.J. (2013). Anorexia nervosa is linked to reduced brain structure in reward and somatosensory regions: A meta-analysis of VBM studies. BMC Psychiatry.

[bib77] Tricomi E., Balleine B.W., O'Doherty J.P. (2009). A specific role for posterior dorsolateral striatum in human habit learning. European Journal of Neuroscience.

[bib78] Trifilieff P., Feng B., Urizar E., Winiger V., Ward R.D., Taylor K.M. (2013). Increasing dopamine D2 receptor expression in the adult nucleus accumbens enhances motivation. Molecular Psychiatry.

[bib79] Wardlaw J.M., Brindle W., Casado A.M., Shuler K., Henderson M., Thomas B. (2012). A systematic review of the utility of 1.5 versus 3 Tesla magnetic resonance brain imaging in clinical practice and research. European Radiology.

[bib80] Watson H.J., Yilmaz Z., Thornton L.M., Hübel C., Coleman J.R.I., Gaspar H.A. (2019). Genome-wide association study identifies eight risk loci and implicates metabo-psychiatric origins for anorexia nervosa. Nature Genetics.

[bib81] Yin H.H., Ostlund S.B., Balleine B.W. (2008). Reward-guided learning beyond dopamine in the nucleus accumbens: The integrative functions of cortico-basal ganglia networks. European Journal of Neuroscience.

[bib82] Zarei M., Mataix-Cols D., Heyman I., Hough M., Doherty J., Burge L. (2011). Changes in gray matter volume and white matter microstructure in adolescents with obsessive-compulsive disorder. Biological Psychiatry.

[bib83] Zigmond A.S., Snaith R.P. (1983). The hospital anxiety and depression scale. Acta Psychiatrica Scandinavica.

