# Supplementary material for: Basal ganglia volume and shape in anorexia nervosa
Source: Appetite. 2020 Jan 1;144:104480. doi: 10.1016/j.appet.2019.104480 (PMC6891247; doi:10.1016/j.appet.2019.104480)
Supplement: Multimedia component 1 [file mmc1.docx]

Supplementary Table 1. Information about types of psychotropic medication

| Group | Name | % (N) of AN participants |
| --- | --- | --- |
| Anti-depressant | Citalopram | 38.46% (N =5) |
|  | Fluoxetine | 38.46% (N = 5) |
|  | Venlafaxine | 7.69% (N = 1) |
| Anti-anxiety | Pregabalin | 7.69% (N = 1) |
| Anti-psychotic | Quetiapine | 7.69% (N = 1) |
| Thyroid medication | Levothyroxine | 7.69% (N = 1) |
| Anti-convulsant | Lamotrigine | 7.69% (N = 1) |

Information regarding the type of psychotropic medication was collected as part of Study 2 (Fonville et al., 2014)

Supplementary Table 2. Subcortical volumes by datasets

| Hemisphere | Volume | Dataset 1  (N = 47) | Dataset 2  (N = 53) | t score, p-value |
| --- | --- | --- | --- | --- |
| Left | Caudate | 3704.43 (331.82) | 3573.46 (370.69) | t(98) = -1.85, p = 0.067 |
|  | Putamen | 4417.65 (423.86) | 4408.18 (557.44) | t(98) = -0.10, p = 0.924 |
|  | Pallidum | 1365.61 (167.86) | 1337.96 (174.64) | t(98) = -0.81, p = 0.423 |
|  | NAcc | 589.01 (103.97) | 578.90 (111.93) | t(98) = -0.47, p = 0.642 |
| Right | Caudate | 3736.25 (351.17) | 3592.53 (381.76) | t(98) = -1.95, p = 0.054 |
|  | Putamen | 4234.60 (420.60) | 4244.60 (549.47) | t(98) = 0.10, p = 0.920 |
|  | Pallidum | 1458.16 (158.24) | 1432.07 (182.08) | t(98) = -0.76, p = 0.449 |
|  | NAcc | 516.26 (95.81) | 511.56 (100.69) | t(98) = 0.50, p = 0.615 |

NAcc = nucleus accumbens

Supplementary Table 3. Correlation between clinical characteristics and subcortical volumes

| Group | Hemisphere | Volume | BMI | EDEQ total | Duration of illness (in years) |
| --- | --- | --- | --- | --- | --- |
| AN | Left | Caudate | ρ = 0.10, p = 0.525 | ρ = 0.08, p = 0.593 | ρ = -0.25, p = 0.118 |
|  |  | Putamen | ρ = 0.28, p = 0.063 | ρ = 0.38, p = 0.010 | ρ = -0.13, p = 0.430 |
|  |  | Pallidum | ρ = 0.01, p = 0.960 | ρ = 0.10, p = 0.515 | ρ = 0.22, p = 0.170 |
|  |  | NAcc | ρ = 0.25, p = 0.100 | ρ = 0.05, p = 0.757 | ρ = 0.14, p = 0.371 |
|  | Right | Caudate | ρ = 0.15, p = 0.297 | ρ = 0.14, p = 0.352 | ρ = -0.17, p = 0.298 |
|  |  | Putamen | ρ = 0.30, p = 0.045 | ρ = 0.26, p = 0.088 | ρ = 0.04, p = 0.826 |
|  |  | Pallidum | ρ = 0.20, p = 0.193 | ρ = 0.36, p = 0.015 | ρ = 0.29, p = 0.062 |
|  |  | NAcc | ρ = 0.12, p = 0.418 | ρ = 0.05, p = 0.760 | ρ = 0.02, p = 0.877 |
| HC | Left | Caudate | ρ = -0.17, p = 0.224 | ρ = -0.18, p = 0.198 | N/A |
|  |  | Putamen | ρ = -0.26, p = 0.056 | ρ = -0.03, p = 0.829 | N/A |
|  |  | Pallidum | ρ = 0.03, p = 0.809 | ρ = -0.26, p = 0.059 | N/A |
|  |  | NAcc | ρ = -0.02, p = 0.908 | ρ = -0.10, p = 0.477 | N/A |
|  | Right | Caudate | ρ = -0.18, p = 0.194 | ρ = -0.10, p = 0.490 | N/A |
|  |  | Putamen | ρ = -0.12, p = 0.377 | ρ = -0.04, p = 0.782 | N/A |
|  |  | Pallidum | ρ = 0.08, p = 0.552 | ρ = -0.12, p = 0.388 | N/A |
|  |  | NAcc | ρ = 0.10, p = 0.473 | ρ = -0.06, p = 0.672 | N/A |

BMI = body mass index, EDEQ = Eating Disorders Examination Questionnaire

|  |  |  |  |  |
| --- | --- | --- | --- | --- |
|  |  |  |  |  |
|  |  |  |  |  |
|  |  |  |  |  |
|  |  |  |  |  |
|  |  |  |  |  |
|  |  |  |  |  |
|  |  |  |  |  |
|  |  |  |  |  |

Supplementary Table 4. Regression between vertex indices and EDEQ total score within the HC group while controlling for dataset.

| Hemisphere | Structure | Index | Voxels | Peak MNI coordinates | | | Peak t score, TFCE corrected p-value |
| --- | --- | --- | --- | --- | --- | --- | --- |
|  |  |  |  | x | y | z |  |
| Left | Putamen | c1 | 95 | -30 | 8 | -4 | t = 5.27, p < 0.0001 |
|  |  | c2 | 42 | -14 | 9 | -5 | t = 5.14, p < 0.0001 |

EDEQ = Eating Disorder Examination Questionnaire, HC = healthy comparison, TFCE = Threshol-free cluster enhancement, MNI = Montreal Neurological Institute
